# The Role of Type I Interferons in Tuberculosis and in Tuberculosis-Risk-Associated Comorbidities

**DOI:** 10.3390/idr17040081

**Published:** 2025-07-08

**Authors:** Florence Mutua, Ruey-Chyi Su, Terry Blake Ball, Sandra Kiazyk

**Affiliations:** 1 Department of Medical Microbiology and Infectious Diseases, Max Rady College of Medicine, University of Manitoba, Room 543-745 Bannatyne Avenue, Winnipeg, MB R3E 0J9, Canada; ruey.c.su@phac-aspc.gc.ca (R.-C.S.); t.blake.ball@phac-aspc.gc.ca (T.B.B.); 2 Department of Medical Microbiology and Immunology, Kenyatta National Hospital Campus, University of Nairobi, Nairobi P.O. Box 30197-00100, Kenya; 3 National Sexually Transmitted and Bloodborne Infections Laboratory Division, JC Wilt Infectious Diseases Research Centre, Public Health Agency of Canada, 1015 Arlington St, Winnipeg, MB R3E 3P6, Canada

**Keywords:** tuberculosis, interferon signature, type I interferon, HIV, diabetes, systemic lupus erythematosus, COVID-19, end-stage renal disease, silicosis

## Abstract

The identification of a type I interferon-induced transcriptomic signature in active tuberculosis suggests a potential role for these interferons in the pathogenesis of tuberculosis. Comorbidities such as human immunodeficiency virus, diabetes, systemic lupus erythematosus, end-stage renal disease, and coronavirus disease are epidemiologically linked to an increased risk for reactivation of latent tuberculosis infection. Notably, type I interferons are also implicated in the pathogenesis of these conditions, with a recognizable type I interferon transcriptomic signature. The mechanisms by which type I interferons in tuberculosis-risk-associated comorbidities may drive the progression of tuberculosis or maintenance of latent infection however remain largely unknown. This review summarizes the existing literature on the increased association between type I interferons, focusing on interferon-α and -β, and the heightened risk of tuberculosis reactivation. It also underscores the similarities in the immunopathogenesis of these comorbidities. A better understanding of these mechanisms is essential to guide the development of host-directed interferon therapies and improving diagnostic biomarkers in *M. tuberculosis* infection.

## 1. Introduction

Tuberculosis (TB), caused by *Mycobacterium tuberculosis*, remains an infectious disease of significant health concern worldwide. Despite its identification as the causative agent of TB over 150 years ago, *M. tuberculosis* still causes more than 10 million active TB cases and over one million deaths annually [1]. TB is one of the top ten causes of death globally and one of the leading infectious causes of mortality [2]. 

The primary mode of transmission of infection is through inhalation of *M. tuberculosis* droplet nuclei, which has been shown to have several potential outcomes. These include clearance of the bacteria via an effective innate immune response, establishment of an asymptomatic latent TB infection (LTBI), or progression to symptomatic active TB [3,4,5,6]. The potential outcomes of LTBI include life-long infection without progression to active disease, referred to as LTBI or TB infection; incipient TB, a more recent addition in which viable bacilli alternate between periods of dormancy and periods of replication; subclinical disease, in which bacilli are viable in the absence of clinical symptoms; and reactivation to active disease, which often occurs in the event of immunosuppression, also referred to as TB disease [3,4,7,8]. 

*M. tuberculosis* is among the most successful human pathogens in terms of its ability to survive and even persist in the presence of a robust immune response [9]. However, despite significant advances in identifying some of the critical immune factors in infection, the understanding of the immunologic events that lead to maintaining LTBI in its dormant state or progressing to reactivation and active TB disease remains unclear. This understanding is of utmost importance for both predicting and preventing reactivation.

Approximately one-quarter of the world’s population has latent *M. tuberculosis* infection [10]. In this regard, individuals with LTBI represent a large reservoir with the potential for progression to active disease and an important target in the journey to meet the goals of the WHO End Tuberculosis strategy [11,12]. Immunocompetent individuals with LTBI have a 5% to 10% lifetime risk of reactivation to active disease, which increases in the presence of comorbidities that compromise the protective immunity to TB [13,14]. The highest increase in risk is observed in those with human immunodeficiency virus (HIV) infection, with a 10-fold or higher increase in risk; risk is also significantly increased among individuals with diabetes mellitus, rheumatic diseases, chronic kidney disease (CKD), and among patients taking immunosuppressive drugs (Table 1) [15,16,17,18,19]. 

The possible mechanisms by which these comorbidities, particularly HIV, drive progression to active TB have been widely investigated [20,21,22,23]. However, the host’s immune response to *M. tuberculosis* is complex and incompletely understood [24]. Recent interrogation of the transcriptomic profiles of *M. tuberculosis*-infected individuals has revealed a type I interferon (IFN)-driven gene signature in active TB, pointing to a role for type I IFN in *M. tuberculosis* disease progression [25,26,27,28,29]. Interestingly, these IFNs have also been linked to the pathogenesis of several autoimmune diseases and infections [30,31]. Further, research has revealed type I IFN-regulated molecular signatures in HIV [32,33], type I diabetes [34,35,36], rheumatic diseases [37,38], and coronavirus disease (COVID-19) [39]. Thus, a feature that is observed in active TB is also observed in diseases associated with an increased risk of progression to active TB. In this review, we present findings from the literature on the role of type I IFNs (IFN-α and IFN-β) in the risk of reactivation of TB, exploring the potential mechanisms by which type I IFNs in these diseases may drive TB reactivation.

**Table 1:** Incidence, risk, and type I IFN involvement of TB-associated comorbidities.

Comorbidity	Global Incidence	TB Risk	Type I IFN-Driven Impacts
HIV/AIDS	1.3 million new infections (39.9 million people living with HIV) [40]	10–100-fold [17]	Type I IFN signature, decline in systemic pDCs, immune activation, reduced T cell frequency and function
Diabetes (both T1D and T2D)	588.7 million adults [41]	2- to 4-fold [41]	Early type I IFN signature, lymphocyte exhaustion, impaired T cell and macrophage function
SLE	5.14 (1.4 to 15.13) per 100,000 person-years [42]	6.11-fold [43,44]	Type I IFN signature, reduced frequency and function of pDCs and macrophages, activation/IFN-driven inflammation
ESRD	4.9–9.7 million [45]	6.9- to 52.5-fold [46]	Increased IFN-α expression
COVID-19/Long COVID	777 million [47]	1.34 [48]	Type I IFN signature, activation/IFN-driven inflammation
Silicosis	138,971 [49]	4.01 [50,51]	IFN-driven inflammation

HIV/AIDS—human immunodeficiency virus/acquired immunodeficiency syndrome; T1D—type 1 diabetes; T2D—type 2 diabetes; SLE—systemic lupus erythematosus; ESRD—end-stage renal disease; COVID—coronavirus disease; pDC—plasmacytoid dendritic cell; IFN—interferon.

## 2. Type I IFNs and *M. tuberculosis* Infection

Type I IFNs are a family of cytokines that consist of IFN-α subtypes, -β, -ε, -k, and -ω. The primary cellular sources of type I IFNs are the innate immune cells, plasmacytoid dendritic cells (pDCs), and macrophages, through the type I IFN pathway. Plasmacytoid DCs (pDCs), the primary producers of type I IFNs, produce 1000-fold more of this IFN on recognition of viral pattern-associated molecular patterns (PAMPs) than that produced by other cells in similar infections [52]. 

### 2.1. M. tuberculosis-Specific Induction of Type I IFNs

**Figure 1:**
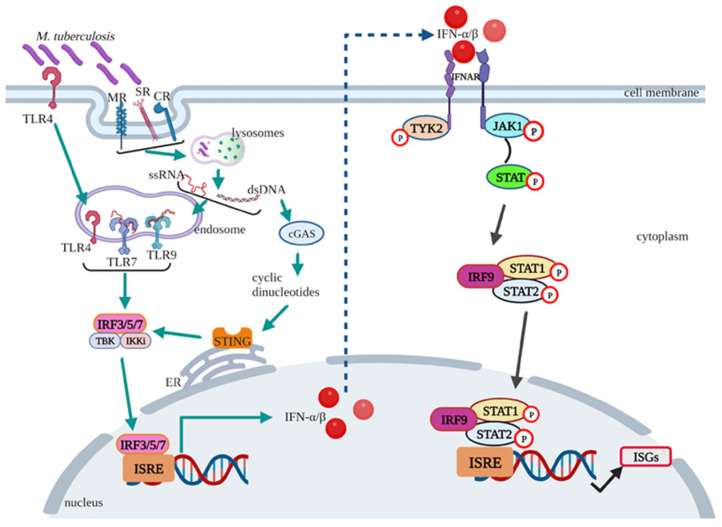
Type I IFN induction and signalling pathway in *M. tuberculosis* infection. The figure illustrates the cascade of type I IFN production following *M. tuberculosis* infection and the subsequent induction of the IFN signalling pathway, resulting in the downstream production of ISGs. TLR—Toll-like receptor; MR—mannose receptor; SR—scavenger receptor; CR—complement receptor; ssRNA—single-stranded ribonucleic acid; dsDNA—double-stranded deoxyribonucleic acid; ER—endoplasmic reticulum; IFN—interferon, IFNAR—interferon-alpha receptor; IRF—interferon regulatory factor; ISRE—interferon-sensitive response element; TYK—tyrosine kinase; JAK—Janus kinase; STAT—signal transducer and activator of transcription; ISG—interferon-stimulated genes; cGAS—cyclic GMP-AMP synthase; STING—stimulator of IFN genes; TBK1—tyrosine kinase 1; IKK—IκB kinase, ISGs—interferon stimulated genes. Created with BioRender.com (Available from https://www.biorender.com/) (Accessed on 30 January 2025).

The recognition of *M. tuberculosis* PAMPs by pattern recognition receptors (PRRs) on the cell surfaces (Toll-like receptor (TLR) 4; mannose receptors (MRs); scavenger receptors (SRs) such as the macrophage receptor with collagenous (MARCO) structure, macrophage scavenger receptor 1 (MSR1), and CD36; and complement receptors (CRs)) or intracellularly in the endosome (TLR7, TLR9, or internalized TLR4) initiates type I IFN production (Figure 1). Activation of cell surface receptors results in the internalization of the receptor into the endosome (TLR4) or in the phagocytosis and degradation of the bacilli (MR, SR, and CR). The latter process results in the release of ssRNA or dsDNA that are recognized by internally expressed TLR7/9. Subsequent secretion of IFN regulatory transcription factors, IRF3 and IRF7, leads to the production of IFN-α and IFN-β [53]. An alternative pathway to type I IFN production involves the DNA motifs, dsDNA, produced from bacterial degradation within phagosomes and released into the cytosol. The DNA is bound by cyclic guanosine monophosphate (GMP)–adenosine monophosphate (AMP) synthase (cGAS) and acts in converting ATP and GTP into cyclic GMP-AMP (cGAMP). cGAMP, a second messenger, binds to the stimulator of IFN genes (STING) located on the membrane of the endoplasmic reticulum, which moves STING to the Golgi apparatus, activating TANK-binding kinase 1 (TBK1) and IκB kinase (IKK). These enzymes activate IRF3 and IRF5, resulting in the production of type I IFNs [53].

The IFNs then bind to IFN-α receptor subunits (IFNAR1 and IFNAR2) and activate Janus kinase 1 (JAK1) and tyrosine kinase 1 (TYK1) to recruit the signal transducer and activator of transcription (STAT) molecules (Figure 1) [54]. The STAT molecules undergo phosphorylation by kinases, form dimers, and bind to IRF9, forming the IFN-stimulated gene factor 3 (ISGF3). This transcription factor complex translocates from the cytoplasm to the nucleus and binds to the IFN-stimulated response elements (ISREs). This binding results in the transcription of IFN-inducible gene expression, leading to the production of type I IFN-stimulated genes (ISGs), the effector molecules of the IFNs [54,55].

### 2.2. Role of Type I IFNs in M. tuberculosis Infection and Pathogenesis

Type I IFNs regulate the expression of a broad range of ISGs which can exert either protective or detrimental effects in the host [56]. A growing body of evidence supports this functional duality of type I IFNs. This is especially evident in viral infections, with antiviral effects observed during acute infections, but they may become immunosuppressive in the chronic phase [57]. In bacterial infections, type I IFNs also demonstrate context-dependent roles, with both beneficial and harmful outcomes reported [58,59]. Although most studies suggest a detrimental effect of type I IFNs in *M. tuberculosis* infection, evidence from mouse models indicates that this duality may also apply in TB, with both protective and pathogenic effects observed depending on the context [56,60,61].

In *M. tuberculosis* infection, the induction of type I IFNs by more virulent strains in comparison to less virulent strains and the dominance of type I IFNs and downstream genes in active TB suggest a pathogenic role [59]. Several mechanisms have been proposed for an immunomodulatory role of type I IFNs in *M. tuberculosis* infection [32,59]. The primary mechanism of pathogenesis of this is thought to be through the induction of an interleukin (IL)-10 anti-inflammatory response [59,62,63]. IL-10, a potent anti-inflammatory cytokine, suppresses and, therefore, impairs the protective Th1 immune responses in *M. tuberculosis* infection. In early infection, IL-10 has been shown to impair the capacity of differentiated T cells to migrate to the lung parenchyma. This leads to the accumulation of CD4+ T cells within the lung vasculature and a reduction in their numbers in the lung parenchyma, resulting in reduced bacterial antigen sensing. The result is increased bacterial proliferation and decreased clearance, leading to disease progression [64,65]. In addition, the IFNs inhibit the production of IL-12, tumour necrosis factor (TNF), and IL-1β in *M. tuberculosis*-infected human monocytes [59,66]. IL-1 is crucial for stimulating host resistance against *M. tuberculosis* by promoting prostaglandin E2 (PGE2) synthesis. Hence, the suppression of IL-1 by type I IFNs limits the production of this eicosanoid, eliminating the regulatory effects of PGE2 on type I IFNs [59,67]. TNF is known to drive the maturation of pDCs incapable of producing IFN-α; therefore, it is essential for controlling type I IFN production. The suppression of TNF production in diseases such as rheumatoid arthritis (RA), which requires anti-TNF treatment, disrupts this equilibrium, leading to the inhibition of DC maturation and the production of excessive amounts of IFN-α/β [68,69]. Type I IFNs also downregulate cell surface IFN-γ receptor (IFNGR) mRNA expression in macrophages, resulting in reduced responsiveness to IFN-γ, a protective cytokine that induces the killing function of macrophages to eliminate infectious pathogens [31,59,70,71,72,73]. Macrophages play a crucial role in controlling *M. tuberculosis* infection, via phagocytosis, intracellular killing, and antigen presentation. Type I IFNs show a paradoxical effect on macrophages in TB. The IFNs cause the death of *M. tuberculosis*-infected macrophages, resulting in the release of the bacilli and allowing for their replication, as well as driving immunopathology [74,75,76]. The IFNs also impair the function of macrophages in phagocytosis and intracellular killing of *M. tuberculosis*, enabling bacillary replication. Studies have shown that type I IFNs influence the polarization of macrophages from an M1 phenotype, which is crucial in controlling *M. tuberculosis* infection, to an M2 phenotype, driving disease progression [77,78]. The macrophage phenotype polarization appears to be related to the presence of IFN-γ. Conversely, in the absence of IFN-γ, type I IFNs inhibit the activation of M2 macrophages, playing a protective role in infection [60].

The various mechanisms by which type I IFNs modulate the immune response in *M. tuberculosis* infection support the notion that they play a crucial role in the pathogenesis of TB. This evidence culminates in the revealing of the IFN signature. Recent research has revealed the presence of a type I IFN-driven gene signature in active TB [25,26,27,28,79]. The term “IFN signature” is used to denote the expression of genes regulated by IFNs. This term is not exclusive to type I IFNs; type II and III IFNs also drive gene expression, and multiple IFNs may regulate similar genes [80]. Aside from its identification in active TB patients, the IFN signature has been shown to correlate with disease severity and to resolve with treatment, making it potentially useful for monitoring treatment response [28,81]. Further, a type I IFN transcriptomic signature has also demonstrated potential for predicting LTBI reactivation [82,83].

## 3. Type I IFNs and TB-Risk-Associated Comorbidities

Type I IFNs are pivotal in driving the pathogenesis of diseases that are known risk factors for TB reactivation in patients with comorbidities. Conditions such as HIV, diabetes, SLE, and other rheumatic diseases clearly illustrate this connection. Recognizing the impact of type I IFNs on these diseases empowers us to better address and manage the complexities of TB reactivation in affected patients. 

### 3.1. TB and HIV

TB is a leading cause of death in people living with HIV [84], and HIV is the number one risk factor for LTBI reactivation [16]. TB-HIV co-infection is associated with an increase in morbidity and mortality compared to that caused by each pathogen individually [85]. The risk for developing active TB is approximately 15–21 times greater in HIV-infected individuals than in those uninfected; the risk is reported to be as high as 100 times greater when compared to the general population [17,86]. This risk increases as CD4+ T cell counts decline [87]. While this risk is reduced with the use of combination antiretroviral therapy (cART) and resulting immune reconstitution, it remains higher in HIV-infected individuals with controlled infection compared to HIV-uninfected individuals [88,89]. 

#### Type I IFNs in HIV

The interaction of HIV with pDCs in both acute and chronic HIV infection drives robust type I IFN production, which triggers the type I IFN signalling pathway [90,91]. In acute infection, type I IFNs produced by stimulation of pDCs play a protective role and primarily act by (i) suppressing viral replication and (ii) interfering with viral spread and survival, thereby controlling the infection [92,93,94]. HIV-infected macrophages in acute HIV infection also produce type I IFNs, which are involved in establishing HIV latency [95,96].

In contrast, during chronic infection, persistent pDC activation and type I IFN production show detrimental effects associated with inflammation and immune activation [97]. The persistent production of type I IFNs exhibits a desensitization effect characterized by defects in IFNα/β receptor expression, IFN-α signalling, and type I ISG expression, which favours increased systemic immune activation [98,99,100]. The desensitization effect is associated with increased HIV viral load and promotes HIV progression [101]. The direct relationship between the amount of ISGs and HIV viremia, as well as the inverse relationship between ISGs and CD4+ T cell counts, further supports this relationship [33]. Although the use of cART reduces HIV replication and viral load and is associated with improved CD4+ T cell counts, the expression of ISGs is not entirely reduced to the level of the uninfected [102]. In addition, persistent high-level type I IFN signalling and ISG expression has been observed in some patients on cART, which may impact recovery from immune activation, enhance viral persistence, and be responsible for maintaining viral reservoirs [102].

**Figure 2:**
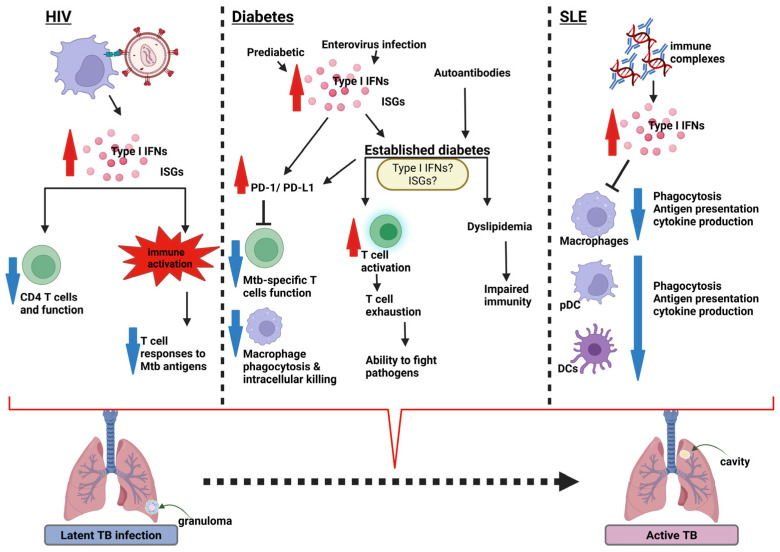
Summary of type I IFN responses in HIV, diabetes, and SLE with potential for reactivation of LTBI. The diagram illustrates the potential mechanisms by which type I IFN-associated diseases can lead to the reactivation of latent tuberculosis infection (LTBI) into active tuberculosis (TB). HIV interacts with pDCs and induces the production of increased levels of type I IFNs. These IFNs act by suppressing CD4 T cell counts and function or by causing immune activation, which suppresses *M. tuberculosis*-specific T cell responses. Diabetes: The production of type I IFNs is induced by either enterovirus infections associated with type 1 diabetes (T1D) or inflammation-mediated T2D. The IFNs act by increasing the PD-1/PD-L1 expression that impairs *M. tuberculosis*-specific function and macrophage phagocytosis and intracellular killing. IFNs also cause T cell exhaustion, impairing their ability to kill pathogens. SLE: Immune complexes and autoantibodies produced in SLE induce increased levels of type I IFNs. The IFNs reduce the capability of macrophages to carry out phagocytosis, antigen presentation, and cytokine production, and that of DCs and pDCs to migrate. The described mechanisms resulting from the type I IFNs in these diseases have the potential, in the presence of LTBI, to drive progression to active TB. Created with BioRender.com (Available from https://www.biorender.com/) (Accessed on 10 June 2025). IFN—interferon; ISGs—interferon stimulated genes; PD—programmed cell death protein; PD-L1—programmed death-ligand 1; DC—dendritic cell; pDC—plasmacytoid dendritic cell; Mtb—*M. tuberculosis*.

These type I IFN-driven dysfunctions may contribute to the increased risk of LTBI reactivation among HIV-positive individuals. The resulting heightened immune activation has been shown to ablate *M. tuberculosis*-specific T cell responses significantly, considered a key element of the protective immune response in LTBI (Figure 2) [98,103,104,105]. In individuals with LTBI, therefore, this type I IFN-rich environment may lead to reactivation and progression to active TB [28,82].

Further studies are needed to elucidate the role of type I IFNs in LTBI reactivation in patients with HIV co-infection to determine the effect of early cART initiation on the residual type I IFN response and to identify new opportunities to intervene and prevent LTBI reactivation.

### 3.2. TB and Diabetes

Although neglected for many years, the association between TB and diabetes has recently re-emerged as a problem due to the increasing prevalence of type 2 diabetes (T2D), especially in developing countries [106,107,108,109]. In 2019, an estimated 15% of adult active TB patients had concurrent diabetes [110,111,112,113]. The convergence of diabetes, LTBI, and active TB creates a storm that may lead to a co-epidemic; this presents an additional challenge to TB control programs, particularly in TB-endemic low- and middle-income countries (LMICs), where both TB and diabetes are endemic, and are resident to an estimated 70% of diabetics [114,115,116,117,118].

Diabetes is associated with a two- to four-fold risk of developing active TB compared to non-diabetic patients. Few studies report on the prevalence of diabetes subtypes, but recent studies have shown that approximately 90% of all diabetic cases are T2D, and 6% are type I diabetes (T1D) [119,120]. This may imply that most LTBI reactivation is associated with T2D, although most studies do not specifically distinguish whether the association is with T1D or T2D. However, there appears to be increased vulnerability for active TB with T1D compared to T2D [121]. The relationship between diabetes and TB is bidirectional; hyperglycemia in diabetes causes immune dysfunction, which hinders the immune response against *M. tuberculosis*, whereas TB and some anti-TB drugs are known to worsen glycemic control [122,123]. 

#### 3.2.1. Type I IFNs in Diabetes

##### Type I Diabetes

There is a paucity of information on the role of type I IFN signalling and ISG profiling in metabolic diseases, but this is an area of growing interest in the field. The identification of increased production of IFN-α in the islet cells of the endocrine pancreas of T1D patients compared to those of non-diabetics was the first hint of a potential role for IFNs in the pathogenesis of T1D [124,125]. Subsequent studies in the early stages of T1D show that IFN-α correlates with the three characteristic features observed in pancreatic islet β cells: increased expression of HLA class 1, increased endoplasmic reticulum (ER) stress markers, and induced apoptosis of β cells [126,127]. These features lead to impaired insulin production observed in T1D. The ER stress regulates the production and sensing of IFN-α induced in response to infections and autoimmunity [128,129]. Increased expression of HLA-1 induces the production of autoantigens and activation of autoreactive cytotoxic CD8 T lymphocytes by B lymphocytes, leading to autoimmunity [127]. Type I IFN signalling in macrophages has also been shown to play a role in the development of diabetes through trafficking of T lymphocytes into the islets [130].

In line with increased Type I IFN production in the development of T1DM, viral infections, characterized by the production of type I IFNs, have also been associated with a predisposition to T1D; this association appears to be particularly strong with enteroviruses [131,132,133,134,135]. These data support the idea that type I IFN levels and increases in type I IFN signatures are essential in the pathogenic processing leading to the development of T1D. There is evidence of elevated levels and increased activity of type I IFNs in the blood and sera of T1D patients, with similar levels observed in both newly diagnosed and previously diagnosed patients [136,137]. This is further supported by the significantly higher quantities of pDCs observed in new-onset T1D patients compared to non-diabetic controls [138]; however, a similar comparison showed lower pDCs in recent-onset T1D patients [139].

In contrast, in established T1D, there is less evidence of sustained type I IFN production. This aligns with the slightly lower pDCs observed in the blood of long-standing T1D patients compared to non-diabetic controls [138,139,140]. Although the ISG signature is absent [35], type I IFN remains linked to several events that are sustained in advanced T1D, which include hyperexpression of HLA molecules on islet cells [141]. This promotes MHC I antigen presentation in pancreatic beta cells, enhancing their visibility to autoreactive CD8+ T cells, which may accelerate T1D progression [142,143]. This hyperexpression could lead to increased immune-mediated damage, further compromising β cell function and insulin secretion. Furthermore, elevated levels of type I IFNs during T1D are associated with lymphocyte exhaustion and increased PD-1 expression, which impairs T cell function and promotes autoimmunity [144]. Additionally, type I IFNs, specifically IFN-α, enhance the expression of PD-L1 on pancreatic β cells [145,146], which limits T cell activity and may contribute to β cell dysfunction. Increased expression of PD-1 in TB inhibits *M. tuberculosis*-specific CD4+ T cell function, macrophage phagocytosis, and intracellular killing contributing to TB reactivation [147].

In contrast to the persistence of the type I IFN transcriptomic signature observed in other IFN-related diseases, HIV and SLE (discussed below), the type I IFN-driven transcriptomic signature in blood from children with a genetic predisposition to T1D is present before the development of autoantibodies. However, the signature appears to resolve after the development of the disease and is therefore absent in established T1D [25,36,148]. Prospective cohort studies and cross-sectional studies suggest that type I IFNs may be involved in the establishment T1D in pre-diabetics [35,36,124]. Evaluation using a reporter cell assay revealed increased serum type I IFN activity in patients with established T1D compared to healthy controls [136]. However, this increased serum activity in diabetes does not appear to translate to the persistence of the IFN signature, which appears to resolve once diabetes develops [35]. A blood-specific signature may not be observed because type 1 diabetes (T1D) is an organ-specific disease, not a systemic one. There are limited human data to indicate a prolonged IFN signature in the blood; however, this is likely due to the compartmentalization of the disease within the islet cells.

##### Type 2 Diabetes

Similar to T1D, the type I IFN signature is also not observed in T2D. However, there is evidence of a role for type I IFNs in the pathogenesis of T2D. It is well known that increased inflammation is observed in T2D [149] and is linked to the activation of STING, the DNA-sensing stimulator of IFN genes, an essential component of the innate immune signalling pathway that governs inflammation-mediated T2D and links the inflammatory and type I IFN pathways [149]. Signalling through the cGAS–cGAMP–STING pathway is known to mediate type I IFN inflammatory responses [130]. Evidence suggests that the STING pathway plays a crucial role in regulating insulin sensitivity in T2D [150].

Type I IFNs play a complex role in the pathogenesis of T2D, influencing immune responses. As in other infections and disease states, their effects can be both protective and detrimental, depending on the context and specific IFN subtype involved. Type I IFNs can inhibit autoimmune processes, as observed in nonobese diabetic (NOD) mice, where specific subtypes reduce diabetes development by modulating the functions of immune cell [151]. Conversely, they can exacerbate T2D by inhibiting IL-10 signalling, which is crucial for regulating T cell responses and maintaining β cell health. This loss of regulation is key to the development of T2D [152]. Therefore, while type I IFNs can offer protective effects against autoimmune diabetes, their chronic presence and specific interactions with other cytokines can lead to detrimental outcomes. Additionally, type I IFNs are postulated to play a role in the dyslipidemia seen in T2D [153,154]. This lipid dysregulation has been shown to increase the risk of infection, including TB [155,156].

It remains unclear what exact role type I IFNs play in the increased susceptibility to TB in diabetic patients. The current literature suggests that type I IFNs in T1D, specifically IFN-α, either upregulate the PD-1/PD-L pathway or cause lipid dysregulation, impairing the immune response against *M. tuberculosis* (Figure 2). The PD-1/PD-L pathway regulates the activation of T cells, driving the cells toward an exhaustion phenotype. The reduced T cell function and inhibition of macrophage phagocytic and intracellular killing processes, which are essential for controlling *M. tuberculosis* infection, are likely links between T1D and an increased risk of LTBI reactivation (Figure 2). Further studies are required to pinpoint the drivers of TB in pre-diabetics and T1D and T2D in both adults and children, taking into consideration geographic and racial differences and comorbidities such as TB.

### 3.3. TB and Rheumatic Diseases

Rheumatic diseases are inflammatory and commonly autoimmune conditions that affect the musculoskeletal system but also cause systemic disease. These include diseases such as systemic lupus erythematosus (SLE), rheumatoid arthritis (RA), and Sjögren’s syndrome (SS).

#### 3.3.1. Type I IFNs in Rheumatic Diseases

Rheumatic diseases exhibit dysregulation of the type I IFN system, characterized by the presence of excess type I IFNs, driven by two factors: genetic variation and receptor activation [37]. Type I IFNs play a significant role in the pathogenesis and clinical management of various rheumatic diseases. Their multifaceted involvement influences immune responses, disease activity, and treatment outcomes. Similar to the other infections and disease states discussed already, in both SLE and RA, type I IFNs modulate immune responses, exacerbating inflammation and autoimmunity [157,158].

#### 3.3.2. TB and SLE

SLE is a chronic autoimmune disease that affects multiple body systems. The pathogenesis of the disease involves the production of self-reactive autoantibodies to autoantigens, such as intracellular proteins and protein–nucleic acid complexes, leading to the formation of circulating immune complexes. The deposition of these complexes in tissues is responsible for damage to various organs, including the skin, kidneys, joints, and lungs [159]. Several studies have identified an increased risk for TB among SLE patients ranging from 5-fold to 15-fold higher risk [160,161,162].

Corticosteroids, including glucocorticoids, are commonly used for SLE and other autoimmune diseases. These drugs directly suppress inflammation [163], and treatment with these drugs alone puts these individuals at an increased risk of TB reactivation [14,17,18,164]. However, studies have also shown that patients with SLE have an increased risk of TB, even when analysis controls for glucocorticoid use. In a study of almost 2000 patients with rheumatic diseases receiving glucocorticoid treatments, patients with SLE had the highest rates of TB reactivation [165]. Similarly, it is likely that in multiple other autoimmune disorders, including Sjögren’s syndrome, myositis, systemic sclerosis, and rheumatoid arthritis, the linkage to type I IFNs in their pathogenesis is directly related to their increased risk for TB.

##### Type I IFNs in SLE

SLE is the prototypic type I IFN-driven disease, primarily driven by IFN-α [166]. Patients present with persistent type I IFN production and a prominent type I IFN-driven signature [167]. Prospective studies have demonstrated an association between this signature and clinical manifestations of SLE [168,169,170]. In addition, treating patients with flares with high-dose glucocorticoids, which block type I IFN production, ablates this signature [51]. 

Characteristic features of SLE include increased cell death through apoptosis, necrosis, and autophagy of immune cells [171,172]. Host nucleic acids released as a result induce the production of autoantibodies and the formation of immune complexes. These complexes stimulate pDCs to produce excessive amounts of type I IFNs, resulting in elevated cytokine levels in the sera of SLE patients.

Sera from SLE patients induce monocyte differentiation into DCs with enhanced antigen-presenting properties, which in turn cause the proliferation of CD4+ T cells, with differentiation primarily driven by IFN-α [173]. Other changes include overactivation of pDCs and myeloid DCs and modification of the chemokine receptor function affecting the migratory capacity of DCs [174,175].

The mechanisms by which SLE increases the risk of TB have not been studied, particularly the role of type I IFNs in a disease driven by IFNs. According to the literature, type I IFNs in SLE appear to reduce the number of macrophages and DCs, thereby impairing phagocytosis, antigen presentation, and cytokine production. These factors suppress the immune response against *M. tuberculosis*, creating an environment that is conducive to reactivation and progression of LTBI (Figure 2) [176,177,178,179,180]. However, the progression to TB in patients on IFN-α blockers also suggests that IFN-α may play a protective role. This warrants further investigation into the role of these IFNs in TB. 

### 3.4. TB and Other TB Risk-Associated Comorbidities with Type I IFN Link

#### 3.4.1. End-Stage Renal Disease (ESRD) and Chronic Kidney Disease (CKD)

There are multiple etiologies of ESRD and CKD, including HIV-associated nephropathy (HIVAN), diabetic nephropathy, and lupus nephritis associated with SLE [181,182]. With the increasing prevalence of diabetes globally, it has become one of the leading causes of ESRD in developed and developing countries [183]. ESRD is the last stage of chronic kidney disease (CKD)—renal failure that requires dialysis. Uremia in ESRD causes immune dysregulation, which predisposes patients to infection [184]. Patients with ESRD have a 6.9- to 52.5-fold higher risk of developing TB than the general population, independent of etiology [46,166,185,186]. The prevalence of LTBI in ESRD patients on dialysis ranges from 20% to 70% [46,168,169,170]. There is also evidence of increased risk for TB in CKD patients, even in those without the need for dialysis [187]. The role of type I IFNs in ESRD has not been clearly defined in all etiologies. However, increased IFN-α levels and expression of IFN-α mRNA in renal biopsies from patients with HIVAN [188] and lupus nephritis [176,189], respectively, suggest a role for type I IFNs in ESRD resulting from these comorbidities. Genes such as APOL1 expressed in HIVAN and lupus nephritis have also been observed in the pathogenic TB signature [177]. It, however, remains unclear whether type I IFNs play any role in the development of TB in diabetic nephropathy. Taken together, the evidence suggests that type I IFNs may play a role in the development of ESRD in HIV, diabetes, and SLE through mechanisms not yet defined. Furthermore, the role that IFNs may play in the development of TB in ESRD has also not been established.

#### 3.4.2. Silicosis

This is an occupational lung disease caused by the inhalation of silica dust. It is a chronic disease with progressive lung inflammation that significantly increases the risk of TB and complicates its treatment and patient outcomes [51,190]. Results from a recent meta-analysis showed a pooled relative risk of TB in silicosis of 4.01 (95% CI: 2.88, 5.58) [51]. The evidence of this risk of TB appears to be in patients with and without a radiological diagnosis of silicosis, although the proof in the latter group of patients is uncertain [51]. Patients with silicotuberculosis show poor treatment outcomes compared to TB patients without silicosis and a higher risk of TB relapse and mortality [191]. In silicosis, exposure to silica particles causes the death of lung cells, resulting in the release of self-dsDNA, which activates a STING-dependent proinflammatory type I IFN response and induces the production of other proinflammatory cytokines [192,193]. Type I IFNs contribute to the characteristic chronic inflammation and fibrosis in the lung [192,193,194]. However, whether these type I IFN-induced effects are related to the risk of TB remains unknown. 

#### 3.4.3. COVID-19

During the COVID-19 pandemic, caused by the SARS-CoV-2 virus, which began in 2019, the disease surpassed TB as the leading cause of death from a single infectious agent, a trend that is now reversing [47]. While a reduction in TB transmission was reported during the pandemic, LTBI reactivation associated with COVID-19 cases was observed, particularly among patients with severe and long COVID-19 [195,196,197,198,199]. Type I IFNs are crucial for controlling SARS-CoV-2 replication during the early stages of infection. Increased IFN-α levels have been demonstrated in COVID-19 patients, particularly in those with severe and long COVID-19 [39,200,201], which may drive LTBI reactivation. However, some studies have reported low levels of type I IFNs but upregulation of ISGs in severe disease associated with delayed or weak IFN responses [196,202,203,204,205,206]. The administration of corticosteroids in severe COVID-19 was associated with good outcomes but was shown to suppress the levels of type I IFNs [207,208]. This type I IFN deficiency is thought to cause T cell exhaustion, particularly in patients with severe COVID-19, presenting another potential mechanism linking COVID-19 and TB reactivation [209,210]. Furthermore, the use of corticosteroids induces immunosuppressive effects that can lead to the reactivation of LTBI [211]. The effects of type I IFN administration in COVID-19 concerning LTBI have not been clearly defined. As a result, it remains uncertain whether the disease itself, the levels of type I IFN, the administration of type I IFNs, or the use of corticosteroids contributed to the reactivation of LTBI. Understanding the role of type I IFNs in the interactions between COVID-19 and TB reactivation is essential.

## 4. Perspectives and Research Opportunities

Infection with *M. tuberculosis* is associated with an annual incidence of more than 10 million cases of active TB and latently infects one in four people globally. The increasing global prevalence of HIV, diabetes, systemic lupus erythematosus (SLE), and end-stage renal disease (ESRD), as well as other comorbidities linked to a higher risk of LTBI reactivation and TB disease, present a significant challenge to TB control efforts. The various comorbidities that increase the risk of TB share a commonality of dysregulated type I IFN responses [212]. This link may reveal important clues to the pathogenesis of TB and lead to the discovery of new avenues to prevent TB reactivation.

Type I interferons (IFNs) play a complex and context-dependent role in TB progression. Numerous studies have identified type I IFN-driven gene expression signatures in active TB, often correlating with disease severity and treatment outcomes [25,26,27,28,29]. These findings suggest a potentially detrimental role for type I IFNs in TB infection and disease progression. Similar to active TB, transcriptional studies show a type I IFN signature persists in chronic HIV and SLE but is downregulated or resolves in established diabetes [35,92,213]. Other functional consequences of type I IFN dysregulation among these comorbidities that may contribute to enhanced TB disease progression include type I IFN-induced immune activation and sustained inflammation; decreased CD4+ T cell counts and impaired function; and reduced number and functionality of macrophages, pDCs, and conventional dendritic cells. These impairments affect key immune processes such as phagocytosis, antigen presentation, and cytokine production. Additionally, type I IFNs may suppress T cell responses, further weakening host defence mechanisms (Figure 2). 

Furthermore, the effects of type I IFNs likely vary depending on the stage of the infection and the host’s immune status. While early type I IFNs may initially control *M. tuberculosis*—especially in the absence of IFN-γ—prolonged exposure may worsen disease outcomes [56,61]. Comorbidities that drive a type I IFN responses may further complicate this dynamic, potentially amplifying harmful immune effects. Additionally, the interplay between comorbidities adds another layer of complexity. For example, antiretroviral therapy use for HIV infection has been linked to metabolic abnormalities including as insulin resistance and T2D [214,215].

### 4.1. Research Priorities to Inform New Intervention Strategies

While disease-driving roles of type I IFNs have been characterized to varying degrees in numerous comorbidities associated with increased risk of TB disease, their specific effects in the context of *M. tuberculosis* infection remain underexplored. In particular, the influence of type I IFNs on LTBI reactivation, progression to active TB, or disease outcomes in the presence of IFN-driven comorbidities is not well defined. Addressing these knowledge gaps is critical to inform the development of host-directed therapies, improve strategies for preventing LTBI reactivation, and support the use of IFN-related biomarkers to identify individuals at highest risk for LTBI reactivation and inform targeted intervention strategies.

Although dysregulated type I IFN responses may promote TB reactivation, they also hold potential as therapeutic tools. Type I IFNs are currently being evaluated as immunomodulatory agents for host-directed therapy for *M. tuberculosis* infection. Clinical studies have shown that administration of INF-α, both aerosolized and subcutaneously, has demonstrated beneficial effects in patients with active TB [216,217]. Intramuscular IFN-α has also been shown to enhance the efficacy of BCG vaccination by promoting *M. tuberculosis*-specific Th1 responses in an in vivo infection model [218]. In a case report, a TB patient with diabetes who was unresponsive to first-line anti-TB treatment showed improvement following the addition of IFN-α-2a [219]. However, type I IFNs should be used cautiously, as treatment with IFN-α and IFN-β has been associated with kidney damage and conditions that may progress to ESRD [220,221,222].

This underscores the importance of monitoring of IFN levels during therapy. Interestingly, although type I IFN-inducible genes are significantly upregulated in active TB compared to healthy controls and LTBI, no significant difference is observed in the concentration of IFN-α and IFN-β between these groups [223]. This discrepancy highlights the limitations of conventional detection methods and underscores the need for more precise tools. Because direct measurement of type I IFNs is challenging, proxies such as ISG quantification and functional assays using functional reporter cell lines (e.g., WISH cells) have been employed [224]. Emerging technologies such as single molecule arrays (SIMOAs) that can give sub-femtomolar sensitivity [225] may soon provide more accurate measurements of type I IFNs in TB and comorbid conditions. These advances could pave the way for improved diagnostics, risk stratification, and therapeutic monitoring.

### 4.2. Concluding Remarks

In summary, we have discussed several comorbidities associated with increased TB risk that also involve dysregulated type I IFN responses in their pathogenesis. The impact of these type I IFNs on the increased risk of LTBI reactivation remains largely unknown and requires further investigation. Targeted treatment of LTBI in high-risk individuals—such as those with HIV, diabetes, chronic kidney disease, or undergoing immunosuppressive therapy—remains a cornerstone of TB control [18]. Improved biomarkers and a deeper understanding of IFN biology could significantly enhance the precision and effectiveness of these interventions.

Ultimately, integrating insights from immunology, clinical research, and emerging technologies will be key to unlocking the full potential of type I IFNs as both biomarkers and therapeutic targets in the global fight against TB.

## Data Availability

Not applicable.

## Abbreviations

The following abbreviations are used in this manuscript:

APOL1	Apolipoprotein 1
cART	Combination antiretroviral therapy
CD	Cluster of differentiation
cGAMP	Cyclic guanosine monophosphate–adenosine monophosphate
cGAS	Cyclic guanosine monophosphate–adenosine monophosphate synthase
CKD	Chronic kidney disease
CR	Complement receptors
DC	Dendritic cells
dsDNA	Double-stranded deoxyribonucleic acid
ER	Endoplasmic reticulum
ESRD	End-stage renal disease
HIV	Human immunodeficiency virus
HIVAN	Human-immunodeficiency-virus-associated nephropathy
HLA	Human leukocyte antigen
IFN	Interferon
IFNAR	Interferon alpha receptor
IFNGR	Interferon gamma receptor
IGRA	Interferon gamma release assays
IKK	IκB kinase
IL	Interleukin
IRF	Interferon regulatory factor
ISG	Interferon-stimulated genes
ISGF	Interferon-stimulated gene factor
ISRE	Interferon-stimulated response element
JAK	Janus kinase
LMICs	Low- and middle-income countries
LTBI	Latent tuberculosis infection
MR	Mannose receptors
NOD	Nonobese diabetic
PAMP	Pattern-associated molecular patterns
PD-1	Programmed cell death protein 1
pDC	Plasmacytoid dendritic cells
PD-L1	Programmed death-ligand 1
PGE2	Prostaglandin E2
PRR	Pattern recognition receptor
RA	Rheumatoid arthritis
RLR	Retinoic-acid-inducible gene I (RIG-I)-like receptors
SIMOA	Single molecule array
SLE	Systemic lupus erythematosus
SR	Scavenger receptor
SS	Sjögren’s syndrome
ssRNA	Single-stranded ribonucleic acid
STAT	Signal transducer and activator of transcription
STING	Stimulator of interferon genes
T1D	Type 1 diabetes
T2D	Type 2 diabetes
TB	Tuberculosis
TBK1	TANK-binding kinase 1
TLR	Toll-like receptor
TNF	Tumour necrosis factor
TST	Tuberculin skin test
TYK	Tyrosine kinase
WHO	World Health Organization
WISH	Wistar Institute Susan Hayflick (a cell line)
